# Supplementary material for: Structural Guidance in 3D Microgel‐in‐Hydrogel Systems to Improve Chondrogenesis
Source: Adv Healthc Mater. 2026 Jul 19;15(29):e71393. doi: 10.1002/adhm.71393 (PMC13447864; doi:10.1002/adhm.71393)
Supplement: Supplementary file 1 [file ADHM-15-0-s001.docx]

Supporting Information

Structural guidance in 3D microgel-in-hydrogel systems to improve chondrogenesis

Julia Kamp^1^, Alisa C. Suturin^1^, Claudia A. Garrido^1^, Xiaole Tong, Jietao Xu, Nicole Kops, Lea-Sophie Müller, Till Hülsmann, Laura Klasen, Tamás Haraszti, Gerjo J. V. M. van Osch, Laura De Laporte*

**Table S1.** Example values for the production of 300 µL the hydrogels with variations in RGD concentration.

|  | 150 µM | 500 µM | 1 mM | 2mM |
| --- | --- | --- | --- | --- |
| Cells | 118.6 µL | 112.0 µL | 102.5 µL | 83.5 µL |
| PEG-VS 4arm, 20k, 20% | 84.4 µL | 87.2 µL | 91.1 µL | 99.0 µL |
| GRGDSPC (25 mg/mL) | 1.7 µL | 5.5 µL | 11.1 µL | 22.2 µL |
| MMP sensitive peptide (3 w/v%) | 95.3 µL | 95.3 µL | 95.3 µL | 95.3 µL |

**Table S2.** Example values for the production of 300 µL of hydrogel with variations in the PEG-VS concentration.

|  | 3.4 wt% | 5 wt% | 6.5 wt% | 10 wt% |
| --- | --- | --- | --- | --- |
| Cells | 172 µL | 130.7 µL | 90.7 µL | 65.9 µL |
| PEG-VS 4arm, 20k, 20% | 59.4 µL | 81.6 µL | 101.1 µL | 146.4 µL |
| GRGDSPC (25 mg/mL) | 19.2 µL | 19.2 µL | 19.2 µL | 19.2 µL |
| MMP sensitive peptide | 49.4 µL (3%) | 68.5 µL (3%) | 89.0 µL (3%) | 68.5 µL (6%) |

**Table S3.** Microgel dimensions specifications

| Microgel size  (µm^3^) | Volume  (µL) | # of microgels per µL hydrogel | Distance between microgels (µm) |
| --- | --- | --- | --- |
| 2.5x2.5x25 | 1.56E -7 | 64 000 | 16.8 |
| 5x5x50 | 1.25 E -6 | 8 000 | 33.7 |
| 10x10x100 | 1.00 E-5 | 1 000 | 67.3 |

**Table S4.** Example values for the production of 300 µL of hydrogel with variations in the microgel size.

|  | 3.4 wt% |
| --- | --- |
| Microgels | 100 µL |
| Cells | 72.0 µL |
| PEG-VS 4arm, 20k, 20% | 59.4 µL |
| GRGDSPC (25 mg/mL) | 19.2 µL |
| MMP sensitive peptide 3% | 49.4 µL |

**Supplementary Information S1.** Description of the magnetic configuration.

The magnetic configuration consists of two coaxial Neodymium Iron Boron (NdFeB) Magnets (Ø35 mm, thickness 5 mm) separated by a 14.25 mm gap. We performed a simulation using the exact magnet geometry of the magnets and the defect size (a cylinder of 4 mm diameter and 8 mm height) positioned centrally between the magnets. The calculated magnetic flux density at the plug midpoint was 254.0 mT. Across the full plug volume, the field ranged from 253.76 to 255.35 mT (mean 254.48 mT; span 1.59 mT, corresponding to 0.63% of the mean), indicating a highly homogeneous field magnitude. In the middle plug plane, the field was even more uniform, with a span of only 0.096% relative to the center value. The local field direction was likewise highly consistent, with a maximum angular deviation of ~0.10° within the plug volume. Because Anisogel alignment has previously been achieved under weak magnetic fields below 100 mT, the present configuration provides a field that is substantially above the relevant alignment regime and can therefore be considered non-limiting for microgel orientation. Any plateau in alignment quality above ~60 mT is thus more likely attributable to practical orientational saturation of the SPION-loaded rods within the gelation window than to insufficient external field strength.

With respect to fabrication limitations, the present geometry demonstrates that for cartilage defect size scale (≤8 mm height), both field strength and homogeneity are sufficient to ensure uniform alignment throughout the volume.

**Figure S1.** Cell alignment in control media in Anisogels with varying microgel dimensions and alignment in optimized PEG-VS hydrogel. A) Schematic protocol of hMSC differentiation in optimized PEG-VS hydrogel droplets for microgels with dimensions of 2.5x2.5x25 µm^3^, 5x5x50 µm^3^, or 10x10x100 µm^3^. B) Representative confocal microscopy images of hMSCs in hydrogels stained with F-actin (green) and DAPI (blue) in the presence of aligned or unaligned microgels (orange). C-D) Representative angle histogram depicting the full width at half maximum (FWHM) of encapsulated cells and aligned (C) or unaligned (D) microgels (10x10x100 µm^3^)

**Figure S2.** Chondrogenic and Osteogenic marker expression for different microgel dimension aligned versus. unaligned in control or chondrogenic conditions for 3 different hMSC donors. Donor 1 (20 year-old female), Donor 2 (19 year-old male) and donor 3 (25 year-old male). Relative expression of A) *SOX9*, B) *ACAN*, C) *COL2A1*, D) *RUNX2*, and E) *COL10A1*. Data are presented as floating bars with a line indicating the mean. A two-way ANOVA was used to evaluate the effects of differentiation and alignment on marker expression. Tukey’s post hoc test was applied for multiple comparisons. Significance is indicated as: 0.1 (ns), 0.05 (*), 0.01 (**), *** (0.001), ****(0.0001).

**Figure S3.** Mechanical characterization of the PEG-based hydrogels and the effect of the embedded microgels. Box plots of the average Young’s modulus of n=50 indentations from n=3 independent gels of Anisogels with three different microgel dimensions (aligned versus unaligned) and a hydrogel without microgels control (A). Representative heatmaps of the surface Young´s modulus obtained by nanoindentation in a 5x5 matrix with 150 µm spacing for hydrogels without microgels (B), 10x10x100 µm3 microgels aligned (C) or unaligned (D).

**Figure S4.** Hematoxylin-eosin staining of osteochondral plugs. Three representative images per condition of retrieved osteochondral plugs from a 6-week *in vivo* mouse orthotopic model representing the worst condition in the top, average in the middle and the best condition in the bottom row.

**Figure S5.** RGB trichrome staining of osteochondral plugs and tissue classification. Classification of the tissue/material in the defect area: A) Cartilage-like tissue stained by Alcian Blue due to glycosaminoglycans, B) bone-like tissue stained by Picrosirius Red due to the mineralized matrix, c) other tissues, sections with unclear morphological characteristics, d) cell infiltrated gel, hydrogel with single cells but not defined tissue formation and e) leftovers or cell free gel. F) Definition of classification in categories.

**Figure S6.** A) Example of an image segmentation of a plug with representative tissue types described in Figure S4. B) Individual quantification of the previously mentioned tissue types using the RGB staining.
